# Effectiveness of confidential reports to physicians on their prescribing of antipsychotic medications in nursing homes

**DOI:** 10.1186/s43058-020-00013-9

**Published:** 2020-02-25

**Authors:** Noah M. Ivers, Monica Taljaard, Vasily Giannakeas, Catherine Reis, Cara L. Mulhall, Jonathan M.C. Lam, Ann N. Burchell, Gerald Lebovic, Susan E. Bronskill

**Affiliations:** 1 Women’s College Research Institute, Women’s College Hospital, 76 Grenville Ave., Toronto, ON M5S 1B2 Canada; 2 ICES, Toronto, Canada; 3 Institute of Health Policy, Management and Evaluation, University of Toronto, Toronto, Canada; 4 Department of Family and Community Medicine, University of Toronto, Toronto, Canada; 5 School of Epidemiology and Public Health, University of Ottawa, Ottawa, Canada; 6 Clinical Epidemiology Program, Ottawa Hospital Research Institute, Ottawa, Canada; 7 Health System Performance, Ontario Health (Quality), Toronto, Canada; 8 Li Ka Shing Knowledge Institute, St. Michael’s Hospital, Toronto, Canada; 9 Sunnybrook Research Institute, Toronto, Canada

**Keywords:** Antipsychotic prescribing, Nursing homes, Interrupted time series, Audit and feedback

## Abstract

**Background:**

Antipsychotic medication use in nursing homes is associated with potential for harms. In Ontario, Canada, an agency of the provincial government offers nursing home physicians quarterly audit and feedback on their antipsychotic prescribing. We compared the characteristics of physicians who did and did not engage with the intervention, and assessed early changes in prescribing.

**Methods:**

This population-level, retrospective cohort study used linked administrative databases to track prescribing practices in nursing homes pre-intervention (baseline), immediately post-initiative (3 months), and at follow-up (6 months). Exposure variables identified whether a physician signed up to participate (or not) or viewed the feedback following sign up (or not). Differences in the proportion of days that residents received antipsychotic medications at 6 months compared to baseline by exposure(s) were assessed using a linear mixed effects regression analysis to adjust for a range of resident, physician, and nursing home factors. Benzodiazepine and statin prescribing were assessed as a balance and tracer measures, respectively.

**Results:**

Of 944 eligible physicians, 210 (22.3%) signed up to recieve the feedback report and 132 (13.9%) viewed their feedback. Physicians who signed up for feedback were more likely to have graduated from a Canadian medical school, work in urban nursing homes, and care for a larger number of residents. The clinical and functional characteristics of residents were similar across physician exposure groups. At 6 months, antipsychotic prescribing had decreased in all exposure groups. Those who viewed their feedback report had a signicantly greater reduction in antipsychotic prescribing than those who did not sign up (0.94% patient-days exposed; 95% CI 0.35 to 1.54%, *p* = 0.002). Trends in prescribing patterns across exposure groups for benzodiazepines and statins were not statistically significant.

**Interpretation:**

Almost a quarter of eligible physicians engaged early in a voluntary audit and feedback intervention related to antipsychotic prescribing in nursing homes. Those who viewed their feedback achieved a small but statistically significant change in prescribing, equivalent to approximately 14,000 fewer days that nursing home residents received antipsychotic medications over 6 months. This study adds to the literature regarding the role of audit and feedback interventions to improve quality of care.

Contributions to the literature

- Audit and feedback is known to have effects that vary widely.
- This paper evaluates a natural experiment with the launch of a province-wide audit and feedback intervention to improve prescribing in nursing homes.
- It shows how these effects depend on engagement with the intervention.
- It also compares physicians that engaged early-on in this voluntary audit and feedback initiative to those who did not, showing some systematic differences that could inform future work targeting clinicians whose patients are most in need of improved care.

## Background

Antipsychotic medications are commonly used in nursing homes, especially in patients with agitation and/or behavioral disturbances [1]. The potential risks of antipsychotic medications in older adults include cardiovascular events, falls, decreased cognition, and mortality [2–7]. For older residents living in nursing homes, risks of unmanaged aggressive behavior must also be considered for those living with, and caring for, the resident [8]. Therefore, the goal for clinicians, nursing homes, and health systems is not complete avoidance of antipsychotic medications but regular reassessment of the balance between risk for harms and benefits.

Health systems have attempted to encourage appropriate antipsychotic medication prescribing through a range of quality improvement strategies [3], including public reporting of potentially inappropriate antipsychotic medication prescribing in nursing homes [9, 10]. These strategies are not consistently effective [2, 3, 11–14]. One challenge arising in the interpretation of such evidence to inform policy is that those individuals willing to participate in trials of quality improvement strategies are not necessarily representative of the target population [15] and would benefit most from the intervention.

Herein, we describe an evaluation of the early impact of a voluntary, large-scale audit and feedback (A&F) initiative on antipsychotic medication prescribing in Ontario nursing homes. A&F works by directing recipients’ attention to a gap between desired and actual practice, so that efforts can be made to close this gap. Just as pills only work for those who take them, A&F is likely to work only for those who engage with the intervention. Our objectives were to describe the extent of early engagement in this initiative across nursing home physicians; compare the characteristics of physicians, nursing homes, and residents by extent of engagement; and assess whether engagement was associated with changes in the proportion of nursing home residents receiving antipsychotics over time.

## Methods

### Study design

**Fig. 1:**
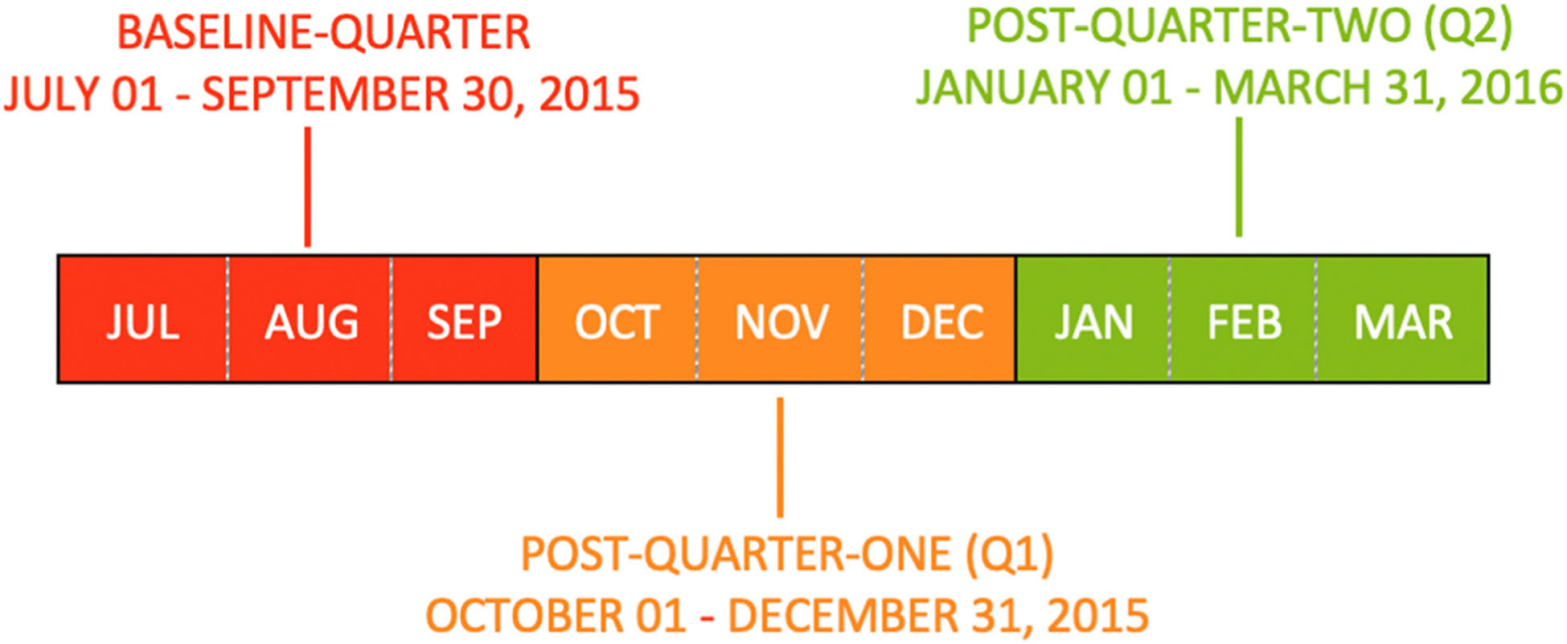
Study timeline

This was a population-level, retrospective cohort study of nursing home residents and their most responsible physicians in Ontario, Canada, from July 2015 to March 2016. This time period covers 3 months preceding the intervention (baseline-quarter, July to September 2015), the immediate post-intervention 3-month period (post-quarter-one (Q1), October to December 2015), and next 3-month period (post-quarter-two (Q2), January to March 2016; see Fig. 1). The study received approval from the Research Ethics Board at Women’s College Hospital.

### Setting

Ontario is Canada’s most populous province, with approximately 13 million people. All personal and nursing care within nursing homes in Ontario is funded by the provincial government through the Ministry of Health and Long-Term Care. Residents are responsible for accommodation charges such as room and board, the costs of which are set by the provincial government and are standard across the province. Rate reductions are available through a government subsidy for those with low income on a case-by-case basis. Prescription drug costs for nursing home residents are covered by the Ontario Drug Benefit program, if prescribed by an Ontario physician or other authorized prescriber. In nursing homes, residents typically have a most responsible physician who prescribes their medications. Day-to-day care is handled by allied health professionals including nurses and personal support workers, with ratios stipulated by provincial legislation.

Health Quality Ontario, now Ontario Health (Quality), is the provincial government agency mandated to monitor and report to the public on the quality of health care provided in Ontario and to support improvements in quality. In 2015, in collaboration with Health Quality Ontario, we established an implementation science laboratory to support the optimization of A&F initiatives in Ontario [16].

### Data sources

Data were obtained from administrative databases linked using encoded identifiers and analyzed at ICES. ICES is a prescribed entity in Ontario with the capacity to hold and link patient-level databases for the purposes of health system evaluation and planning (the research team could not alter these records). The databases at ICES include information on all hospital and nursing home admissions in the province, all visits to emergency departments, physician billing claims, and vital statistics, as well as prescription data for those covered under the provincial health insurance program [17–21]. Intervention exposure data were captured by Health Quality Ontario and shared confidentially for analysis at ICES (www.ices.on.ca). ICES is an independent, non-profit research institute whose legal status under Ontario’s health information privacy law allows it to collect and analyze health care and demographic data, without consent, for health system evaluation and improvement. This project was approved by ICES’ Privacy and Legal Office. It was also approved by local research ethics boards at Women’s College Hospital and the University of Toronto.

### Cohort development

Nursing home residents aged 66 to 105 were eligible for inclusion in the cohort if they were admitted to a nursing home in Ontario at any time between July 1, 2015, and March 31, 2016. A resident could leave and re-enter the cohort if they were discharged (i.e., for a hospitalization) and then readmitted to a nursing home at a later date within this period. Residents remained in the cohort until their discharge date, death date, or end of the observation period. The Continuing Care Reporting System-Long-Term Care was used to assess date of admission and discharge, as well as demographic, clinical, and functional data, captured through the validated Resident Assessment Instrument (RAI) [22]. A full RAI assessment completed by nursing home staff is legislatively mandated within 14 days of admission and updated annually or with a change in status; a quarterly RAI assessment is required every 92 days. For each 3-month period under investigation, residents were assigned to a most responsible physician according to previously defined algorithms [10]. We excluded patients whose most responsible physicians could not benefit from the intervention due to data suppression in the feedback reports (i.e., physicians with fewer than six nursing home residents have their data suppressed for privacy reasons (due to small cell sizes)).

### Baseline physician, nursing home, and resident characteristics

We extracted characteristics from the administrative databases during the baseline-quarter (i.e., July to September, 2015), using the earliest month of data available during this time period. We used the ICES physician database to assess prescriber characteristics, including sex, age, years in practice, specialty, and foreign medical graduate status. We assessed the number of residents for whom each physician was the most responsible provider (Additional file 1). We also assessed total Ontario Health Insurance Program (OHIP) billings to describe the number of nursing home claims in each time period, and the proportion of total resident assessments this represented of the physician’s entire nursing home practice. For nursing homes, we used the institutional facilities database at ICES to assess nursing home characteristics, namely the number of beds, rurality, and private/public ownership status.

We used the RAI data to ascertain demographic and clinical characteristics of residents that might be associated with the outcomes of interest, including sex, age, duration of residency in the home, comorbid conditions (e.g., Alzheimer’s (including other dementia), depression), and clinical assessment scores (e.g., activity of daily living scale, pain scale, depression rating score, likelihood of falls scale, aggressive behavior score). We used OHIP data to determine whether residents had a specialist consultation in the prior year by a geriatrician or psychiatrist. We also used OHIP to assess whether the resident had any physician encounters with a recorded diagnosis of psychosis in the prior 5 years. We used the Canadian Institute for Health Information (CIHI) datasets to assess whether residents had an emergency department visit in the prior year (using the National Ambulatory Care Reporting System (NACRS) database) and whether residents had a hospital admission in the prior year (using the Discharge Abstract Database (DAD)). These databases provide complete population-level data for the variables of interest.

### Intervention and engagement

The Health Quality Ontario reports for physicians working in nursing homes were initially developed as part of a broader Appropriate Prescribing Demonstration Project, in partnership with the Ontario Medical Association and the provincial government [23]. The reports were developed with input from a multidisciplinary team of experts and stakeholders including nursing home physicians [24]. Health Quality Ontario uses administrative data sources to report on a series of quality indicators, and physicians across the province can sign up to receive confidential information about their practice. The reports are updated and re-released quarterly. (In this study, we examined effects related to the initial report released on September 29, 2015, and the subsequent two reports released on January 29, 2016, and April 29, 2016. See Additional file 2 for examples of the reports).

Beginning in July 2015, Health Quality Ontario promoted the reports to nursing home physicians via communication materials distributed by Health Quality Ontario and external partners (including the Ontario Long-Term Care Association, the Ontario Association of Non-Profit Home and Services for Seniors, and the Ontario Long-Term Care Clinicians). To sign up for the report, physicians had to provide consent to receive the report, and verify their email address and identity. When a new report was available for download, those who signed up would receive email notification from Health Quality Ontario. To view the report, physicians had to log into their account via Health Quality Ontario’s secure web portal, and then download a PDF of the report. The steps required to engage with the reports created three natural levels of exposure for our analysis: (1) physicians who did not sign up during the study period, (2) physicians who signed up but did not view the report(s) during the study period, and (3) physicians who viewed at least one report.

### Prescribing outcomes

The primary outcome was the proportion of days a resident was prescribed any antipsychotic medication. The Ontario Drug Benefit database holds complete, population-level dispensing for Ontarians living in nursing homes. For each 3-month time period analyzed (i.e., baseline, Q1, Q2), we obtained the total number of days that the resident was present in the nursing home setting (denominator) and also assessed whether they had at least one active prescription for an antipsychotic that covered those days (numerator). Similar measures were calculated for benzodiazepine prescribing (used as a balance measure to test whether initiatives to decrease antipsychotic medications might result in these high-risk sedative agents being used as an alternative) and statin prescribing (used as a “tracer” or negative control measure, to assess general trends in (de)prescribing habits unlikely to be attributable to the intervention).

### Analysis

Descriptive statistics were used to examine physician, nursing home, and resident characteristics based on patterns of sign up and viewing of reports.

We used histograms to visually inspect the normality of distributions for each outcome measure. The unit of analysis was the individual resident. We used linear mixed effects regression analysis to compare the prescribing outcomes between the three groups from baseline to Q1 and Q2. For this analysis, we excluded those physicians who signed up too late to receive the initial report. The dependent variable was the percentage of nursing home days the resident had an active prescription (i.e., the days covered by the prescription divided by the days in the study period). The exposure variable was a three-level categorical variable, defined as did not sign up, signed up but did not view the report, and signed up and viewed the report. The model included a categorical variable for quarter and the interaction between exposure group and quarter. The correlation in quarterly repeated measures on the same resident was accommodated by specifying an unstructured covariance matrix. A random intercept and random period effect were specified to account for correlation amongst multiple residents nested in the same nursing home and over time.

The model adjusted for the following home-, provider-, and resident-level characteristics: number of beds, urban vs. rural location, and private vs. public nursing home; provider sex, age, years practicing, foreign vs. domestic graduate, number of nursing home residents in the practice, number of nursing homes practicing in, and proportion of OHIP billings in nursing home in comparison to all other billings; and resident sex, age, length of time in nursing home, number of Charlson comorbidities, RAI variables (including diabetes, hypertension, arteriosclerotic heart disease, chronic heart failure, peripheral vascular disease, deep vein thrombosis, cardiac dysrhythmia, dementia, cancer, obstructive air disease, depression, arthritis, Parkinson’s disease), level of function (activities of daily living scale), pain score, depression rating score, likelihood of falls scale, aggressive behavior scale, frailty index, emergency department visits in past year, inpatient hospitalizations in past year, any psychiatric consult in past year, any geriatric consult in past year, and any concurrent benzodiazepine use. Adjusted least square mean differences together with 95% confidence intervals were obtained from the model to estimate differences for all variables (a) between the three exposure groups at baseline, Q1, and Q2; (b) within the three groups from baseline to Q1 and baseline to Q2; and (c) between the three exposure groups in their change from baseline to Q1 and baseline to Q2.

All analyses were conducted using SAS Version 9.4. Given the risk of type 1 error, we a priori selected a *p* value threshold of 0.01 to assess for statistical significance.

## Results

**Fig. 2:**
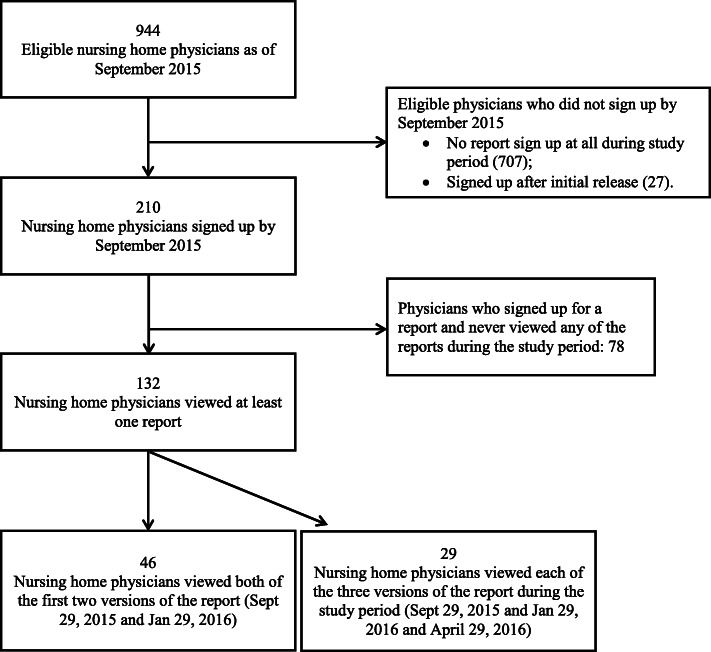
Cohort creation flow diagram

Figure 2 describes the study flow for included patients and their most responsible nursing home physician. In each quarter (i.e., 3-month time period) under analysis, 99.3% of included residents had a unique primary physician prescriber in their nursing home.

### Comparing exposure groups

Table 1 shows the baseline physician, nursing home, and resident characteristics by physician exposure (i.e., sign-up status). A total of 944 physicians met eligibility criteria for the time period of the analysis. Of the 239 physicians who ultimately signed up for the intervention, 2 were not eligible at that time, leaving 237 physicians who signed up for this comparison.

**Table 1:** Baseline characteristics of physicians, nursing homes, and residents in Ontario, by patterns of signing up for a provincial audit and feedback initiative

Variable	Physicians who did not sign up, *n* = 707	Physicians who signed up, *n* = 237	Total, *n* = 944	*p* value
Physician characteristics				
Sex				
Female	181 (25.6%)	59 (24.9%)	240 (25.4%)	0.83
Male	526 (74.4%)	178 (75.1%)	704 (74.6%)	
Age (mean ± SD)	57.60 ± 11.16	56.98 ± 10.66	57.45 ± 11.03	0.45
Years practicing (mean ± SD)	30.93 ± 12.06	30.40 ± 11.37	30.80 ± 11.89	0.546
Medical graduate location				
Foreign graduate	149 (21.1%)	34 (14.3%)	183 (19.4%)	0.02
Canadian graduate	558 (78.9%)	203 (85.7%)	761 (80.6%)	
Number of LTC residents per month (mean ± SD)	49.63 ± 53.38	66.35 ± 57.77	53.83 ± 54.97	*< .001*
Number of LTC billings per month (mean ± SD)	88.54 ± 114.37	127.72 ± 162.45	98.49 ± 129.33	*< .001*
Percent of total billings in month in LTC (mean ± SD)	17.65 ± 24.22	26.87 ± 28.40	19.99 ± 25.65	*< .001*
Nursing home characteristics				
Number of beds in primary LTC home (mean ± SD)	128 (88–179)	150 (97–200)	128 (90–189)	*< .001*
Setting of primary LTC home				
Urban	539 (76.2%)	201 (84.8%)	740 (78.4%)	*0.01*
Rural	168 (23.8%)	36 (15.2%)	204 (21.6%)	
Ownership status of primary LTC home				
Non-profit	329 (46.5%)	114 (48.1%)	443 (46.9%)	0.346
Profit	372 (52.6%)	123 (51.9%)	495 (52.4%)	
Unknown	6 (0.8%)	0 (0.0%)	6 (0.6%)	
Resident characteristics	*n* = 35,091	*n* = 15,726	*n* = 50,817	
Sex				
Female	25,335 (72.2%)	11,308 (71.9%)	36,643 (72.1%)	0.498
Male	9756 (27.8%)	4418 (28.1%)	14,174 (27.9%)	
Age (mean ± SD)	86.4 ± 7.5	86.4 ± 7.5	86.4 ± 7.5	0.731
Time in LTC (cat.)				
< 1 year	7223 (20.6%)	3370 (21.4%)	10,593 (20.8%)	0.03
1–4 years	18,746 (53.4%)	8387 (53.3%)	27,133 (53.4%)	
5–9 years	7386 (21.0%)	3167 (20.1%)	10,553 (20.8%)	
10+ years	1736 (4.9%)	802 (5.1%)	2538 (5.0%)	
Charlson comorbidity score (mean ± SD)	0.9 ± 1.5	0.9 ± 1.4	0.9 ± 1.5	0.233
Diabetes*	9413 (26.8%)	4060 (25.8%)	13,473 (26.5%)	0.017
Hypertension*	23,137 (65.9%)	10,320 (65.6%)	33,457 (65.8%)	0.495
Arteriosclerotic heart disease*	5501 (15.7%)	2294 (14.6%)	7795 (15.3%)	*0.002*
Congestive heart failure*	3886 (11.1%)	1648 (10.5%)	5534 (10.9%)	0.047
Peripheral vascular disease*	1935 (5.5%)	892 (5.7%)	2827 (5.6%)	0.473
Deep vein thrombosis*	447 (1.3%)	196 (1.2%)	643 (1.3%)	0.798
Cardiac dysrhythmia*	2449 (7.0%)	1148 (7.3%)	3597 (7.1%)	0.192
Alzheimer’s or dementia*	24,057 (68.6%)	11,018 (70.1%)	35,075 (69.0%)	*< .001*
Cancer*	2950 (8.4%)	1306 (8.3%)	4256 (8.4%)	0.701
Obstructive airway disease*	6073 (17.3%)	2674 (17.0%)	8747 (17.2%)	0.403
Depression*	11,793 (33.6%)	5168 (32.9%)	16,961 (33.4%)	0.1
Psychosis*	1603 (4.6%)	725 (4.6%)	2328 (4.6%)	0.834
Arthritis*	480 (1.4%)	238 (1.5%)	718 (1.4%)	0.199
Parkinson’s disease*	2533 (7.2%)	1156 (7.4%)	3689 (7.3%)	0.595
Level of function (activities of daily living)* (mean ± SD)	16.9 ± 7.2	16.9 ± 7.4	16.9 ± 7.3	0.603
Pain score* (mean ± SD)	0.4 ± 0.7	0.4 ± 0.7	0.4 ± 0.7	0.901
Depression rating score* (mean ± SD)	2.0 ± 2.4	2.1 ± 2.4	2.0 ± 2.4	*< .001*
Likelihood of falls scale*				
Low risk of falls	29,535 (84.2%)	13,116 (83.4%)	42,651 (83.9%)	*0.03*
Medium/high risk of falls	5556 (15.8%)	2610 (16.6%)	8166 (16.1%)	
Aggressive behavior scale* (mean ± SD)	1.4 ± 2.1	1.5 ± 2.3	1.4 ± 2.2	*< .001*
Frailty index*				
Robust (score < 0.2)	5319 (15.2%)	2385 (15.2%)	7704 (15.2%)	0.307
Pre-frail (score = 0.2 to 0.3)	10,773 (30.7%)	4725 (30.0%)	15,498 (30.5%)	
Frail (score > 0.3)	18,999 (54.1%)	8616 (54.8%)	27,615 (54.3%)	
ED visits in past year	12,862 (36.7%)	5570 (35.4%)	18,432 (36.3%)	0.007
Mean ± SD	0.7 ± 1.4	0.7 ± 1.4	0.7 ± 1.4	0.127
Any inpatient hospitalizations in past year	6520 (18.6%)	2861 (18.2%)	9381 (18.5%)	0.298
Any psychiatric consult in past year	4498 (12.8%)	2029 (12.9%)	6527 (12.8%)	0.793
Any geriatric consult in past year	2031 (5.8%)	922 (5.9%)	2953 (5.8%)	0.738
Any antipsychotic use	9596 (27.3%)	4178 (26.6%)	13,774 (27.1%)	0.068
Any statin use	6209 (17.7%)	2551 (16.2%)	8760 (17.2%)	*< .001*
Any benzodiazepine use	4359 (12.4%)	1809 (11.5%)	6168 (12.1%)	*0.003*

*IQR* interquartile range, *LTC* long-term care

*Captured from the most recent Resident Assessment Instrument data

Physicians who signed up for the report were more likely to work in larger (average of 162 beds (SD 89.4)), urban nursing homes (78.4%). These physicians were more likely to have graduated medical school in Canada and tended to have a greater proportion of the practice focused on nursing home care, with a larger nursing home resident load. The average charactersistics of residents in each practice did not differ between the physicians who did and did not sign up for the report, although those who signed up had slightly greater proportions of their patient group with a history of Alzheimer’s, depression, aggressive behavior, and elevated fall risk. There was no statistically significant difference in baseline antipsychotic medication prescribing rates between those who did and did not sign up to receive a report, but those who signed up prescribed benzodiazepines and statins to a smaller proportion of their roster.

Table 2 describes the same characteristics mentioned above for comparing exposure groups, focusing on those who did and did not view their reports. Of the 210 physicians who signed up in time to receive the initial intervention, 132 viewed at least 1 report and 78 did not view any of their reports. These groups were quite similar on the measured characteristics, although Canadian medical graduates viewed their reports more often than foreign medical graduates, and slightly fewer residents of physicians who viewed their reports received psychiatric consultations in the prior year.

**Table 2:** Baseline physician and resident characteristics by report view

Variable	Physicians with no report views, *n* = 78	Physicians with at least one report view, *n* = 132	Total, *n* = 210	*p* value
Physician characteristics				
Sex				
Female	18 (23.1%)	36 (27.3%)	54 (25.7%)	0.501
Male	60 (76.9%)	96 (72.3%)	156 (74.3%)	
Age (mean ± SD)	55.7 ± 10.1	53.7 ± 11.1	54.4 ± 10.8	0.204
Years practicing (mean ± SD)	28.9 ± 10.9	27.1 ± 11.9	28 (20–36)	0.284
Medical graduate location				
Foreign graduate	16 (20.5%)	11 (8.3%)	27 (12.9%)	*0.01*
Canadian graduate	62 (79.5%)	121 (91.7%)	183 (87.1%)	
Number of LTC residents per month (mean ± SD)	59.7 ± 55.4	63.8 ± 54.1	62.3 ± 54.5	0.602
Number of LTC billings per month (mean ± SD)	138.8 ± 195.7	109.8 ± 114.6	120.6 ± 150.1	0.176
Percent of total billings in month in LTC (mean ± SD)	20.2 ± 23.6	25.4 ± 29.8	23.4 ± 27.7	0.191
Nursing home characteristics				
Number of beds in primary LTC home (mean ± SD)	169.2 ± 102.1	160.9 ± 77.6	164.0 ± 87.4	0.503
Setting of primary LTC home				
Urban	69 (88.5%)	108 (81.8%)	177 (84.3%)	0.201
Rural	9 (11.5%)	24 (18.2%)	33 (15.7%)	
Ownership status of primary LTC home				
Non-profit	38 (48.7%)	67 (50.8%)	105 (50.0%)	0.775
Profit	40 (51.3%)	65 (49.2%)	105 (50.0%)	
Resident characteristics	*n* = 8758	*n* = 15,427	*n* = 24,185	
Sex				
Female	6144 (70.2%)	10,632 (68.9%)	16,776 (69.4%)	0.045
Male	2614 (29.8%)	4795 (31.1%)	7409 (30.6%)	
Age (mean ± SD)	85.8 ± 7.5	85.3 ± 7.5	85.5 ± 7.5	*< .001*
Time in LTC (cat.)				
< 1 year	4502 (51.4%)	7721 (50.0%)	12,223 (50.5%)	0.082
1–4 years	3070 (35.1%)	5455 (35.4%)	8525 (35.2%)	
5–9 years	1001 (11.4%)	1887 (12.2%)	2888 (11.9%)	
10+ years	185 (2.1%)	364 (2.4%)	549 (2.3%)	
Charlson comorbidity score (mean ± SD)	1.2 ± 1.7	1.2 ± 1.6	1.2 ± 1.7	0.041
Diabetes*	2280 (26.0%)	3964 (25.7%)	6244 (25.8%)	0.564
Hypertension*	5775 (65.9%)	9917 (64.3%)	15,692 (64.9%)	*0.01*
Arteriosclerotic heart disease*	1238 (14.1%)	2197 (14.2%)	3435 (14.2%)	0.821
Congestive heart failure*	1132 (12.9%)	1776 (11.5%)	2908 (12.0%)	*0.001*
Peripheral vascular disease*	526 (6.0%)	893 (5.8%)	1419 (5.9%)	0.489
Deep vein thrombosis*	113 (1.3%)	171 (1.1%)	284 (1.2%)	0.207
Cardiac dysrhythmia*	692 (7.9%)	1091 (7.1%)	1783 (7.4%)	0.018
Alzheimer’s or dementia*	15,965 (66.0%)	5685 (64.9%)	10,280 (66.6%)	*0.007*
Cancer*	822 (9.4%)	1276 (8.3%)	2098 (8.7%)	*0.003*
Obstructive airway disease*	1583 (18.1%)	2651 (17.2%)	4234 (17.5%)	0.08
Depression*	2556 (29.2%)	4231 (27.4%)	6787 (28.1%)	*0.003*
Psychosis*	431 (4.9%)	795 (5.2%)	1226 (5.1%)	0.429
Arthritis*	139 (1.6%)	204 (1.3%)	343 (1.4%)	0.094
Parkinson’s disease*	665 (7.6%)	1065 (6.9%)	1730 (7.2%)	0.046
Level of function* (activities of daily living) (mean ± SD)	15.8 ± 7.3	15.6 ± 7.5	15.7 ± 7.4	0.039
Pain score* (mean ± SD)	0.5 ± 0.8	0.5 ± 0.7	0.5 ± 0.8	*< .001*
Depression rating score* (mean ± SD)	1.9 ± 2.3	1.9 ± 2.2	1.9 ± 2.3	0.143
Likelihood of falls scale*				
Low risk of falls	7137 (81.5%)	12,618 (81.8%)	19,755 (81.7%)	0.562
Medium/high risk of falls	1621 (18.5%)	2809 (18.2%)	4430 (18.3%)	
Aggressive behavior scale* (mean ± SD)	1.4 ± 2.2	1.4 ± 2.2	1.4 ± 2.2	0.18
Frailty index*				
Robust (score ≤ 0.2)	1550 (17.7%)	2894 (18.8%)	4444 (18.4%)	0.112
Pre-Frail (score = 0.2 to 0.3)	2964 (33.8%)	5192 (33.7%)	8156 (33.7%)	
Frail (score ≥ 0.3)	4244 (48.5%)	7341 (47.6%)	11,585 (47.9%)	
ER visits in past year	4945 (56.5%)	8475 (54.9%)	13,420 (55.5%)	0.022
Any inpatient hospitalization	3403 (38.9%)	5749 (37.3%)	9152 (37.8%)	0.014
Any psychiatric consult in past year	1435 (16.4%)	2241 (14.5%)	3676 (15.2%)	*< .001*
Any geriatric consult in past year	1218 (13.9%)	2060 (13.4%)	3278 (13.6%)	0.226
Any antipsychotic use	2663 (30.4%)	4792 (31.1%)	7455 (30.8%)	0.288
Any statin use	1796 (20.5%)	3154 (20.4%)	4950 (20.5%)	0.908
Any benzodiazepine use	1077 (12.3%)	1936 (12.5%)	3013 (12.5%)	0.568

*IQR* interquartile range, *LTC* long-term care

*Captured from the most recent Resident Assessment Instrument data

### Changes in prescribing patterns

**Fig. 3:**
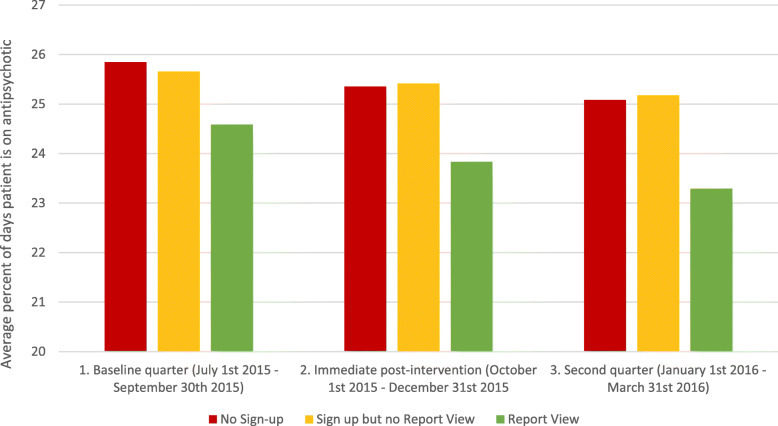
Adjusted antipsychotic prescribing at each time period, by exposure group. Adjusted for nursing home variables (number of beds, urban vs. rural location, private vs. public institution), physician variables (sex, age, years practicing, Canadian vs. foreign graduate, number of nursing home residents, number of nursing home institutions practicing, percent of billings in nursing homes), and resident characteristics (sex, age, time in nursing home, Charlson comorbidity scale, diabetes, hypertension, arteriosclerotic heart disease, heart failure, peripheral vascular disease, deep vein thrombosis, cardiac dysrhythmia, Alzheimer’s, dementia, cancer, obstructive airway disease, depression, arthritis, Parkinson’s disease, activities of daily living scale, pain score, depression rating scale, likelihood of falls scale, aggressive behavior scale, frailty index, emergency department visits in past year, inpatient hospitalizations in past year, any phychiatric consult in past year, any geriatric consult in past year, any benzodiazepine use)

**Fig. 4:**
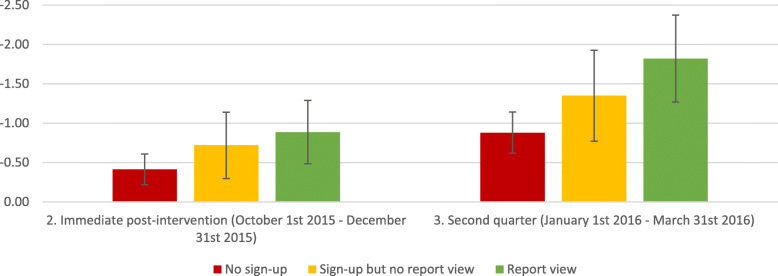
Adjusted difference in percentage of days patient is on antipsychotic, relative to baseline-quarter. Adjusted for nursing home variables (number of beds, urban vs. rural location, private vs. public institution), physician variables (sex, age, years practicing, Canadian vs. foreign graduate, number of nursing home residents, number of nursing home institutions practicing, percent of billings in nursing homes), and resident characteristics (sex, age, time in nursing home, Charlson comorbidity scale, diabetes, hypertension, arteriosclerotic heart disease, heart failure, peripheral vascular disease, deep vein thrombosis, cardiac dysrhythmia, Alzheimer’s, dementia, cancer, obstructive airway disease, depression, arthritis, Parkinson’s disease, activities of daily living scale, pain score, depression rating scale, likelihood of falls scale, aggressive behavior scale, frailty index, emergency department visits in past year, inpatient hospitalizations in past year, any psychiatric consult in past year, any geriatric consult in past year, any benzodiazepine use)

Figure 3 describes the model-adjusted output for the mean percentage of days receiving antipsychotic medications for each exposure group at baseline, Q1, and Q2, illustrating differences in prescribing over time for those physicians who viewed the reports, but not for other groups of physicians. Figure 4 depicts the model-adjusted change in prescribing at each timepoint, relative to the baseline value. Additional file 3: Figures S1 and S2 depict the model-adjusted change for the balance measure, the percentage of days on benzodiazepines, and for the tracer measure, the percentage of days on statins. Together, these figures illustrate change over time in prescribing for each group of physicians.

Table 3 quantifies the changes in prescribing for these medication classes over time within each exposure group. We observed significant changes over time in all three groups for antipsychotic medications. The greatest reduction in antipsychotic medication over time was observed for the group that viewed at least one of their reports: − 1.82% (95% CI − 1.27 to − 2.37%; *p* < 0.0001).

**Table 3:** Prescription rates: within-group changes over time

Time	Did not sign up, *n* = 707	Signed up but did not view, *n* = 78	Signed up and viewed report, *n* = 132
Mean percentage of nursing home days on antipsychotics			
Baseline	25.8	26.3	25.0
3 months	25.4	25.6	24.1
6 months	24.9	24.9	23.2
Within-group changes over time (*p* value)	< 0.0001	< 0.0001	< 0.0001
Change from baseline to 3 months: adjusted least square mean (95% CI)	− 0.41 (− 0.61, − 0.22)	− 0.72 (− 1.14, − 0.30)	− 0.89 (− 1.29, − 0.48)
Change from baseline to 6 months: adjusted least square mean (95% CI)	− 0.88 (− 1.14, − 0.62)	− 1.35 (− 1.93, − 0.77)	− 1.82 (− 2.37, − 1.27)
Mean percentage of nursing home days on benzodiazepines^			
Baseline	10.5	9.8	10.2
3 months	10.4	9.6	10.0
6 months	10.3	9.7	9.7
Within-group changes over time (*p* value)	0.001	0.387	0.001
Change from baseline to 3 months: adjusted least square mean (95% CI)	− 0.10 (− 0.21, 0.02)	− 0.18 (− 0.45, 0.08)	− 0.16 (− 0.41, 0.09)
Change from baseline to 3 months: adjusted least square mean (95% CI)	− 0.25 (− 0.38, − 0.12)	− 0.12 (− 0.41, 0.17)	− 0.52 (− 0.80, − 0.25)
Mean percentage of nursing home days on statins			
Baseline	17.5	16.4	16.5
3 months	16.9	15.8	15.8
6 months	16.2	15.2	15.0
Within-group changes over time (*p* value)	< 0.0001	< 0.0001	< 0.0001
Change from baseline to 3 months: adjusted least square mean (95% CI)	− 0.54 (− 0.64, − 0.44)	− 0.54 (− 0.76, − 0.32)	− 0.61 (− 0.82, − 0.40)
Change from baseline to 3 months: adjusted least square mean (95% CI)	− 1.20 (− 1.35, − 1.05)	− 1.17 (− 1.51, − 0.84)	− 1.44 (− 1.76, − 1.12)

Multivariable linear mixed effects regression adjusted for the following: nursing home variables (number of beds, urban vs. rural location, private vs. public institution), physician variables (sex, age [cont.], years practicing [cont.], Canadian vs. foreign graduate, number of nursing home residents [cont.], number of nursing home institutions practicing in [cont.], percent of billings in nursing homes), and resident characteristics (sex, age [cont.] time in nursing home [cont.], Charlson comorbidity scale [cont.], diabetes, hypertension, arteriosclerotic heart disease, heart failure, peripheral vascular disease, deep vein thrombosis, cardiac dysrhythmia, Alzheimer’s, dementia, cancer, obstructive airway disease, depression, arthritis, Parkinson’s disease, activities of daily living scale, pain score, depression rating scale, likelihood of falls scale, aggressive behavior scale, frailty index, emergency room visits in past year [cont.], inpatient hospitalizations in past year [cont.], any psychiatric consult in past year, any geriatric consult in past year, any benzodiazepine use). Analyses restricted to physicians who signed up in time for the initial release of the intervention

^Any benzodiazepine use dropped from adjustment in this model

Table 4 summarizes the comparisons across exposure groups for changes in prescribing over time. For antipsychotic medications, there was a statistically significant difference between the group that viewed the report and the group that did not sign up at all (0.94% greater reduction; 95% CI 0.35 to 1.54%; *p* = 0.002). For both benzodiazepines and statins, no statistically significant changes in prescribing for these classes over time were observed when comparing those who viewed the report and those did not sign up.

**Table 4:** Prescription rates: pairwise comparisons for changes over 6 months from baseline

Change	Adjusted least square mean difference (% scale)	95% confidence interval	*p* value
Mean percentage of nursing home days on antipsychotics			
Report view vs. no sign up	− 0.94	− 1.54, − 0.35	*0.002*
No report view vs. no sign up	− 0.47	− 1.09, 0.15	0.137
Report view vs. no report view	− 0.47	− 1.26, 0.31	0.239
Mean percentage of nursing home days on benzodiazepines*			
Report view vs. no sign up	− 0.27	− 0.57, 0.02	0.071
No report view vs. no sign up	0.12	− 0.19, 0.44	0.433
Report view vs. no report view	− 0.40	− 0.79, 0.00	0.048
Mean percentage of nursing home days on statins			
Report view vs. no sign up	− 0.24	− 0.58, 0.10	0.171
No report view vs. no sign up	0.02	− 0.33, 0.38	0.893
Report view vs. no report view	− 0.26	− 0.72, 0.19	0.254

Multivariable linear mixed effects regression adjusted for the following: nursing home variables (number of beds, urban vs. rural location, private vs. public institution), physician variables (sex, age [cont.], years practicing [cont.], Canadian vs. foreign graduate, number of nursing home residents [cont.], number of nursing home institutions practicing in [cont.], percent of billings in nursing homes), and resident characteristics (sex, age [cont.] time in nursing home [cont.], Charlson comorbidity scale [cont.], diabetes, hypertension, arteriosclerotic heart disease, heart failure, peripheral vascular disease, deep vein thrombosis, cardiac dysrhythmia, Alzheimer’s, dementia, cancer, obstructive airway disease, depression, arthritis, Parkinson’s disease, activities of daily living scale, pain score, depression rating scale, likelihood of falls scale, aggressive behavior scale, frailty index, emergency room visits in past year [cont.], inpatient hospitalizations in past year [cont.], any psychiatric consult in past year, any geriatric consult in past year, any benzodiazepine use). Analyses restricted to physicians who signed up in time for the initial release of the intervention

*Any benzodiazepine use dropped from adjustment in this model

## Discussion

### Main findings

In this observational study, we found that total exposure to antipsychotic medications amongst nursing home residents declined over time and the rate of decline was associated with greater engagement in a voluntary A&F intervention. Specifically, physicians who both signed up and then viewed their personalized prescribing reports had a greater decrease in their prescribing rates than physicians who did not. The decrease in prescribing over 6 months amongst the group that viewed their A&F reports equates to approximately 14,000 fewer days that any nursing home resident was exposed to antipsychotic medications in that timeframe. In contrast, we did not observe changes in prescribing over time in other drug classes that the intervention did not address.

Only 12.5% of eligible physicians fully engaged with this voluntary A&F initiative during the first 6 months of its availability. Interestingly, the physicians who engaged with the A&F initiative were already slightly less likely to prescribe antipsychotics at baseline, suggesting a latent interest in the topic. Our analysis identifies that certain characteristics were associated with physicians who voluntarily engaged. Physicians working in larger urban nursing homes and for whom nursing home residents represented a greater proportion of their practice seemed most likely to engage. This suggests relatively successful recruitment of higher-volume physicians. We also found that foreign medical graduates were less likely to sign up for and view the reports. Other studies examining physician characteristics associated with low-value care have also identified that foreign medical graduates may be more likely to over-test or over-treat [25]. This may reflect differences in social networks between early adopters and relative laggards [26] as it relates to engagement with data to inform practice. Since prescriber characteristics are associated with antipsychotic medication prescribing independent of resident and nursing home characteristics, an adaptable approach to implementation interventions that allows for recipient customization may be beneficial [27]. Prior research has shown that antipsychotic medication prescribing in Ontario nursing homes may be even more strongly associated with home-level characteristics than prescriber characteristics [2]. This, along with our finding of variable uptake for this provider-focused intervention, indicates a potential role for organizational- and system-level initiatives alongside provider interventions.

### Implications

Prior research indicates that A&F can be effective, especially for prescribing [28], but the extent of effectiveness varies with the characteristics of the intervention [16]. Much research has focused of late on optimizing the design features of feedback [29]. Yet, regardless of how carefully designed the intervention is, feedback cannot be effective if the intended recipient does not engage. Our findings build upon prior work in Ontario indicating that many physicians do not actively engage in existing A&F initiatives [30–34]. Immediate clinical tasks may take priority, and many physicians, whether working in teams or independently, are struggling to keep up rather than looking for ways to get ahead [35]. It is possible that engagement will increase over time as the intervention matures, but it would appear that further research is needed to support both engagement with and action upon clinical performance data.

To address the barrier between signing up and viewing, Health Quality Ontario now sends the reports as email attachments (obviating the need to login to the password-protected website and manually download the report). In addition, since the time of this analysis, sign up rates have increased to over 400 physicians working in nursing homes (plus about 3000 physicians working in office-based primary care). This greater engagement over time reflects an important issue for this study—we purposefully analyzed the initial release of the report to understand early uptake and impact. Future research is needed to understand why some physicians were more likely to engage early in voluntary A&F interventions and how to leverage this information to increase the spread, scale, and impact of A&F and other implementation interventions.

### Limitations

A number of additional caveats must also be highlighted in this study in terms of interpreting the effects on prescribing. First, although we adjusted statistically for measurable confounders, the non-experimental approach cannot be used to attribute causality with confidence; there may be additional confounders we could not capture. Those who voluntarily engaged in the intervention may be different from those who do not in ways that influence prescribing but cannot be captured using administrative data. These include the staffing models in the homes, the use of physical restraints or other techniques (whether appropriate or otherwise) to manage behavioral challenges, and the clinical rationale (whether appropriate or otherwise) for using antipsychotics.

While the lack of effects seen in the tracer and balance outcomes supports conclusions regarding reductions in inappropriate antipsychotic prescribing, it is possible that other compensatory changes in prescribing occurred [36]. Second, the outcome, while objectively and reliably measured independently from the intervention, represents dispensing, not actual pill-taking. Indeed, all the measures used in this study that leveraged routinely collected administrative data were not created to answer the research question posed herein. Fortunately, the risk of measurement bias arising from this should be non-differential across exposure groups. Third, we examined prescribing in three quarterly intervals (one pre- and two post-intervention quarters), using multivariable linear random effects regression with the individual resident nested within homes as the unit of analysis. An alternative approach utilizing additional pre-intervention measures in smaller time intervals (e.g., monthly) could have strengthened our ability to draw causal inferences, but would have required us to make additional model assumptions about the nature of the pre-intervention trend and type of intervention effect, as well as the type of correlation structures over time. Fourth, the data do not permit exploration of practice models that incorporate non-physicians, including nurse practitioners, as the prescriber. Likewise, the feedback was directed solely at physicians. In a team-based environment such as nursing homes, there may be a role for data that supports changes in processes for all team members, as appropriate. Finally, the methodological approach cannot explain why or how changes occurred, or whether initial changes in prescribing were sustained. Three to 6 months appears to be enough time to observe initial changes, but further research is needed to understand how the effects of this sort of intervention may vary over time (i.e., learning and decay effects).

## Conclusion

In summary, we used population data and objective outcomes to pragmatically assess the early effects of a real-world initiative, finding that amongst those who engaged with the intervention, a statistically significant reduction was achieved. We explored the key implementation outcome of engagement with the intervention and identified variation in characteristics across those who did and did not engage. Just as drugs do not work in people who do not take them, A&F cannot work if recipients do not fully engage with their data. When it comes to A&F, the adage “if you build it, they will come” simply does not apply. It would appear that in a context where physicians are independent and autonomous contractors, facilitating engagement in quality improvement must be viewed as a long-term project.

## Supplementary information

**Additional file 1.** Assigning patients to a most responsible physician.

**Additional file 2.** Intervention details: Health Quality Ontario’s MyPractice: Long-Term Care Reports.

**Additional file 3: Figure S1.** Difference in percentage of days patient is on benzodiazepine, relative to baseline quarter. **Figure S2.** Difference in percentage of days patient is on statin, relative to baseline quarter.

## Data Availability

Data access are governed by the policies at ICES. The authors would be happy to share the SAS code if desired.

## Abbreviations

A&F: Audit and feedback
CIHI: Canadian Institute for Health Information
DAD: Discharge Abstract Database
ICES: Institute for Clinical Evaluative Sciences
NACRS: National Ambulatory Care Reporting System
OHIP: Ontario Health Insurance Program
Q1: Post-quarter-one
Q2: Post-quarter-two
RAI: Resident Assessment Instrument
