# Supplementary material for: Effectiveness of confidential reports to physicians on their prescribing of antipsychotic medications in nursing homes
Source: Implement Sci Commun. 2020 Feb 25;1:30. doi: 10.1186/s43058-020-00013-9 (PMC7427908; doi:10.1186/s43058-020-00013-9)
Supplement: Supplementary file 1 — Additional file 1. Assigning patients to a most responsible physician. [file 43058_2020_13_MOESM1_ESM.docx]

**Supplementary File 1 - Assigning patients to a most responsible physician**

Beginning on the first day of a month at least 30 days from admission, and for each following month, we assigned a most responsible physician prescriber based on rostering data (if a resident is denoted in the Client Agency Program Enrolment (CAPE) database held at ICES as enrolled at a nursing home under the care of a specific physician). For residents not formally enrolled into a physician’s practice, we ‘virtually rostered’ by querying the Ontario Health Insurance Program database (OHIP), assigning to a physician who billed a special monthly nursing home management code for the month of interest. Where no such billing code was used, we assigned the resident to the family physician/general practitioner with the greatest total physician billing codes for the month of interest. Finally, where no OHIP billing codes were applied for a resident in a given month, we carried forward the most responsible physician from the prior month.

Using this approach, 34,242 (34.9%) patients were assigned based on rostering data, 63,755 (65.1%) patients were assigned using billing data. Only 9,162 (9.35%) patients changed their most responsible physician during the study.
