# Supplementary material for: Effectiveness of confidential reports to physicians on their prescribing of antipsychotic medications in nursing homes
Source: Implement Sci Commun. 2020 Feb 25;1:30. doi: 10.1186/s43058-020-00013-9 (PMC7427908; doi:10.1186/s43058-020-00013-9)

**Supplementary File 2 – Intervention details: Health Quality Ontario’s MyPractice: Long-Term Care Reports**

The reports for physicians working in nursing homes are known as the *MyPractice*: Long-Term Care report (<http://www.hqontario.ca/Quality-Improvement/Guides-Tools-and-Practice-Reports/Long-Term-Care>). The initial report is the focus of this study; this included aggregated data to describe the physician’s nursing home resident population, and compared the recipient to the Ontario average on four antipsychotic prescribing quality indicators: overall rate; new starts; continuous use; and antipsychotic polypharmacy. They also included ‘change ideas’ or suggested action plans that recipients could carry out to improve the metrics in the report. The prescribing data in the reports was six months old, meaning the September 2015 reports contained data as of March 31, 2015.


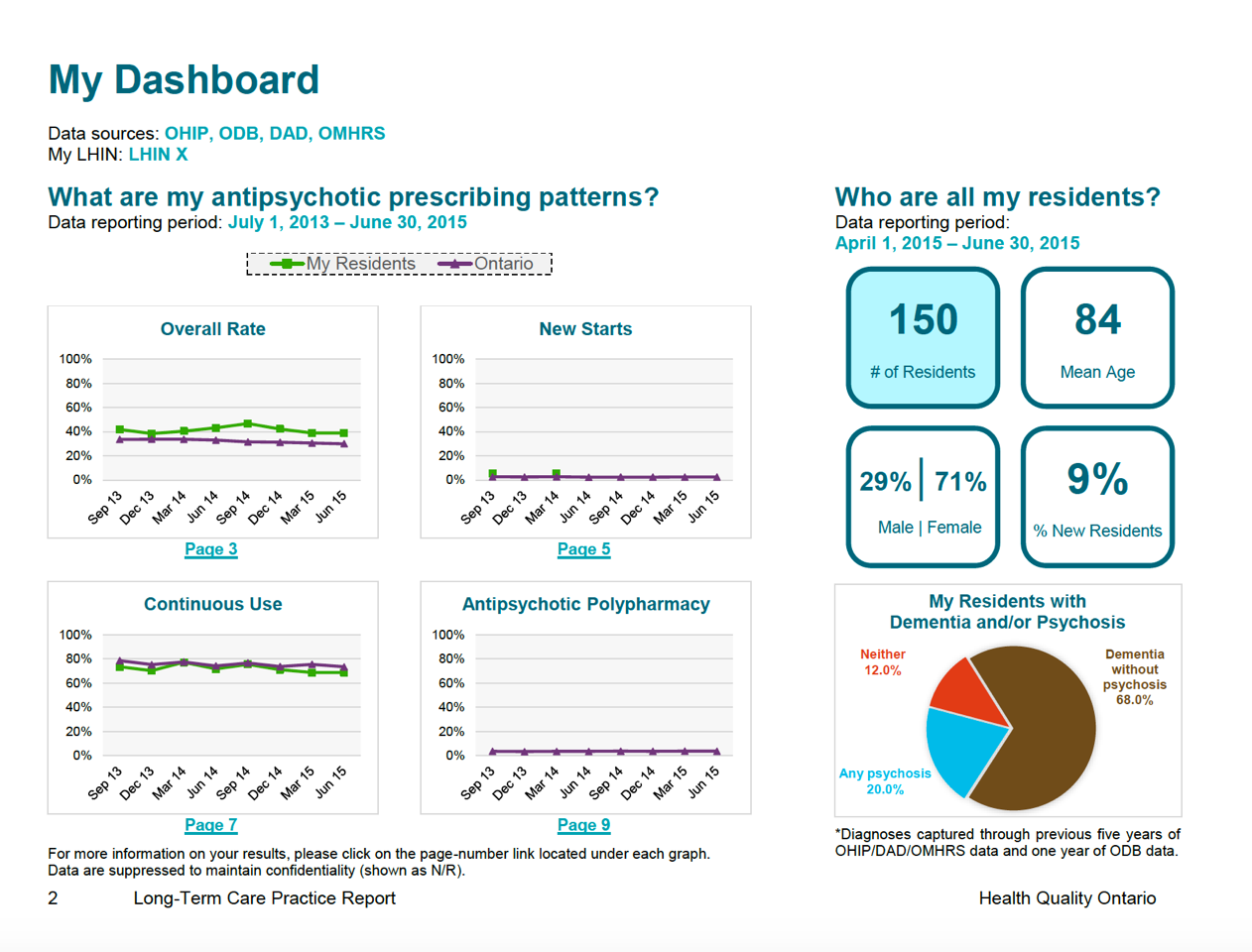

Supplement: Supplementary file 2 — Additional file 2. Intervention details: Health Quality Ontario’s MyPractice: Long-Term Care Reports. [file 43058_2020_13_MOESM2_ESM.docx]
