# Supplementary material for: Effectiveness of confidential reports to physicians on their prescribing of antipsychotic medications in nursing homes
Source: Implement Sci Commun. 2020 Feb 25;1:30. doi: 10.1186/s43058-020-00013-9 (PMC7427908; doi:10.1186/s43058-020-00013-9)
Supplement: Supplementary file 3 — Additional file 3: Figure S1. Difference in percentage of days patient is on benzodiazepine, relative to baseline quarter. Figure S2. Difference in percentage of days patient is on statin, relative to baseline quarter. [file 43058_2020_13_MOESM3_ESM.docx]

**Supplementary File 3 – Additional Figures**

**Figure S1. Difference in percentage of days patient is on benzodiazepine, relative to baseline quarter*^.**

**Figure S2. Difference in percentage of days patient is on statin, relative to baseline quarter*.**

Adjusted for nursing home variables (number of beds, urban vs. rural location, private vs. public institution), physician variables (sex, age, years practicing, canadian vs. foreign graduate, number of nursing home residents, number of nursing home institutions practicing, percent of billings in nursing homes), and resident characteristics (sex, age, time in nursing home, charlson comorbidity scale, diabetes, hypertension, arteriosclerotic heart disease, heart failure, peripheral vascular disease, deep vein throbosis, cardiac dysrhythmia, alzheimers, dementia, cancer, obstructive airway disease, depression, arthritis, Parkinson's disease, activities of daily living scale, pain score, depression rating scale, likelihood of falls scale, aggressive behavior scale, frailty index, emergency department visits in past year, inpatient hospitalizations in past year, any phychiatric consult in past year, any geriatric consult in past year, any benzodiazepine use).]

^Any benzodiazepine use dropped from adjustment in this model.
